# Data From the File Drawer: Bridging the Data Gap About Female First-Year Learners’ Performance and Psychological Well-Being in a Bilingual Context

**DOI:** 10.5334/jopd.141

**Published:** 2026-09-11

**Authors:** Maura Pilotti, Maryam Bo Julaia, Arifi Waked, Omar El-Moussa

**Affiliations:** 1 Prince Mohammad bin Fahd University, SA

**Keywords:** female first-year undergraduate students, prospective and retrospective memory, anxiety, self-efficacy, Middle East

## Abstract

Findings of educational science are too often defined by the contribution of student populations of the Global North. This imbalance is particularly notable in an understudied population of female first-year learners from a country that has only recently emerged from a strict patriarchal order. For these students, the freshman year is considerably challenging, as it requires countering persisting gender stereotypes. This dataset (https://doi.org/10.7910/DVN/KT8UTE) offers a snapshot of their functioning. It contains demographic information, self-reported memory functioning, anxiety, and self-efficacy. Data can enrich existing data sets, thereby contributing to the development of prediction models inclusive of human diversity, and, more broadly, to cross-cultural research.

## (1) Background

The development of the present dataset is motivated by the scarce availability of evidence illustrating cognitive, affective, and behavioral data from student populations of the Global South (Chankseliani & Sopromadze, 2023; Demeter, 2020; Heller & Leeder, 2025). Evidence has often been assumed to uniformly percolate to populations around the globe, even though variability in cultural backgrounds, instructional modes, and contents may suggest otherwise. When such populations are included in research involving educational matters and social, cognitive, and behavioral phenomena, it is usually in the context of institutions located in the Global North (Creighton, 2007). Particularly neglected is the collection of data involving the academic success and well-being of young women from societies emerging from patriarchy, such as Saudi Arabia. Misperceptions of such women plague the legacy media of the Western world.

For female Saudi Arabian undergraduate students, the first year of university studies in either STEM (Science, Technology, Engineering, and Mathematics) or non-STEM programs is considerably challenging. It requires a host of adjustments to academic demands that conflict with persisting gender stereotypes. The latter relegate women to the home and see them as undeserving of professional accomplishments, which once benefited exclusively men. Yet, little is known of Saudi Arabian undergraduate students’ first-year performance at this critical point in their educational and professional journey. The present dataset offers a snapshot of their functioning during the first year of STEM or non-STEM university studies. It specifically focuses on indices of performance before university enrollment (high-school grade point average, GPA) and after the first year of enrollment (first-year GPA). In addition, the dataset relies on self-reports to collect information about students’ confidence in their abilities, their emotional difficulties (e.g., the severity of anxiety symptoms and their impact on areas of daily activities), and cognitive challenges (e.g., concerns about memory functioning, under the assumption that memory is the common denominator of academic tasks). To do so, participants complete three questionnaires, each assessing a different construct: anxiety symptoms, memory concerns, and academic confidence. Students are organized into two fields: STEM (computer science and engineering) and non-STEM fields (business and law). The STEM fields of computer science and engineering are specifically selected because these are fields in which women are substantially underrepresented (Creighton, 2007; Kayan-Fadlelmula et al., 2022). Data from programs in these fields offer a platform for examining whether, even in the early stages of Saudi Arabian female learners’ undergraduate educational journey, differences exist between STEM and non-STEM enrollees (Eltoum & Abdelsalam, 2025; Pilotti et al., 2024).

## (2) Methods

### 2.1 Study design

A cross-sectional design was utilized. Data were collected in person from first-year undergraduate students of Saudi Arabian descent enrolled in introductory communication courses of the general education curriculum.

### 2.2 Time of data collection

August 2023 – April 2025

### 2.3 Location of data collection

Prince Mohammad bin Fahd University, Eastern Province, Saudi Arabia

### 2.4 Sampling, sample, and data collection

Data were collected over a period of two years from 1141 female first-year students (mean age 20.56; range 18–28 years) enrolled in the introductory communication courses of a U.S.-based general education curriculum. The reason for choosing these general education courses for participant selection was that they are compulsory for all students, irrespective of their major. Thus, they created a unified performance baseline for the basic skills (e.g., reading, writing, speaking, and critical thinking) expected of first-year students before enrolling in major-related courses. Participants were selected through purposive sampling. If they reported to be of Saudi Arabian descent and had completed their previous education (elementary school and high school) in the country, they were asked to participate. Informed consent was collected prior to participation. It is important to note here that purposive sampling might have limited the generalizability of the present data set to other female students in the Arabian Gulf region.

Recruitment relied on instructors who announced the study and described its purpose and activities during class meetings. Self-reports were collected toward the end of students’ first year of enrollment. Students were Arabic-English bilingual speakers. The minimal English competency required for university enrollment was that of a ‘competent user of the English language’ (as per IELTS standards). Participants included 482 students enrolled in STEM programs (computer science or engineering) and 659 enrolled in non-STEM programs (business and law). These programs were chosen for their ubiquity among Saudi Arabian universities. Furthermore, the STEM fields of computer science and engineering were specifically targeted for their notable underrepresentation of female students. Thus, the selected programs allowed for an examination of whether, even in the early stages of undergraduate students’ educational journey, differences exist between STEM and non-STEM female learners (Pilotti et al., 2024). We did not gather additional information, such as cultural orientation (Hofstede & Bond, 1984), to ensure that the completion of the study would not be excessively taxing on students’ spare time.

Participation occurred at the end of the students’ second semester. The participation rate was 97% (i.e., out of 1176 who were invited). Students completed the questionnaires independently in classrooms, towards the end of class meetings, under the supervision of a researcher to ensure a quiet setting without distractions. Participants accessed questionnaires (Google Forms) electronically through their laptops. All items of each questionnaire required an answer. Students completed the study in one setting (approximately 15–20 minutes). No compensation was offered. Data regarding their first-year GPA were collected after students completed a year of enrollment to ensure a performance assessment that would cover the entire students’ first year of academic activities. First-year GPA included communication courses compulsory for all majors.

### 2.5 Materials/ Survey Instruments

The survey administered to students collected minimal demographic information to foster students’ trust, thereby maximizing response rates and data quality: students’ current age and major (STEM = 1; non-STEM = 0). Besides providing demographic information, students were expected to respond to a set of three self-report measures: The Prospective and Retrospective Memory Questionnaire (PRMQ; Crawford et al., 2003; Smith et al., 2000), the Generalized Anxiety Disorder (GAD-7) scale (Spitzer et al., 2006), and the General Academic Self-Efficacy (GASE) scale (Lindstrøm & Sharma, 2011; Nielsen et al., 2017).

PRMQ (Crawford et al., 2003; Smith et al., 2000) measured students’ concerns about remembering intentions (prospective memory) or recalling previously encoded information (retrospective memory). The questionnaire contained 16 statements of memory lapses, each to be rated on a Likert scale from ‘never’ (1) to ‘very often’ (5). GAD-7 (Spitzer et al., 2006) measured students’ frequency with which anxiety symptoms (7 items) were experienced through a Likert scale ranging from ‘not at all’ (0) to ‘nearly every day’ (3). Thus, the severity with which anxiety was experienced could range from 0 to 21. Furthermore, students indicated the impact of anxiety on a scale from ‘not difficult at all’ (0) to ‘extremely difficult’ (3). Namely, they reported the extent to which anxiety made it difficult for them to perform activities in three different areas of everyday functioning: school, home, and social settings. Lastly, GASE (Lindstrøm & Sharma, 2011; Nielsen et al., 2017) measured students’ confidence in their abilities to perform academic tasks through 5 items, each to be assessed on a 5-point scale ranging from ‘strongly disagree’ (1) to ‘strongly agree’ (5).

High-school GPA and first-year GPA were provided by the Office of the Registrar of the hosting institution. For the computation of first-year GPA (i.e., average of the grades obtained from general education courses), the following scale was used for each completed course: A+: 4.00; A: 3.75; B+: 3.50; B: 3.00; C+: 2.50; C: 2.00; D/D+: 1.00–1.50; and F: 1.49-0. Weighted high-school GPA (including scientific and humanities scores) was computed as a percentage based on the 0–5 scale. All data collected were anonymized after the matching of different sources (questionnaires and institutional data) had taken place.

### 2.6 Quality Control

For PRMQ, Cronbach’s Alpha was 0.83 (prospective memory concern subscale: items 1, 3, 5, 7, 10, 12, 14, and 16) and 0.81 (retrospective memory concern subscale: items 2, 4, 6, 8, 9, 11, 13, and 15). For GAD-7, Cronbach’s Alpha was 0.91 (severity of anxiety), and for GASE, it was 0.80.

During pilot work, test-retest reliability was assessed for all self-report measures by having a subgroup of students (*n* = 35) retake the questionnaires within an interval of approximately two months. Pearson correlation coefficients (*r)* ranged from 0.97 to 0.98 (*p* < 0.05).

The face validity (Nevo, 1995) of each of the self-report scales was assessed through reliance on a sample of students (*n* = 25) from the same population of the current study. Each item was rated on a 5-point scale from relevant (+2) to irrelevant (–2) to the construct intended to be tested. A definition of the construct relevant to each questionnaire (i.e., prospective and retrospective memory, academic self-efficacy, and anxiety) was provided to and discussed with the raters to ensure adequate comprehension before performing the rating task. No item of the PRMQ, GAD-7, and GASE received a rating below +1. The inter-rater agreement was measured through the intraclass correlation: ICC (two-way mixed, absolute agreement, average measurement) values were ≥ 0.88. None of the students who participated in the quality control activities was included in the dataset.

### 2.7 Data anonymization and ethical issues

The dataset is posted on https://doi.org/10.7910/DVN/KT8UTE under the title ‘Bridging the data gap’. The file is in Excel and CSV formats for easy use. All data collected were anonymized after the matching of different sources took place. Informed consent, conforming to APA guidelines, was collected prior to participation. The Deanship of Research IRB of Prince Mohammad bin Fahd University approved the study (PMU-DoR-2023-2024-20) as conforming to the guidelines of ethical research proposed by the Office for Human Research Protections of the U.S. Department of Health and Human Services.

### 2.8 Existing use of data

Until the current date, there are no publications that have originated from this dataset.

## (3) Dataset description and access

### 3.1 Repository location

The dataset is posted on https://doi.org/10.7910/DVN/KT8UTE.

### 3.2 Object/file name

Bridging the data gap. CVS

Bridging the data gap. xlsx

### 3.3 Data type

Primary data

### 3.4 Format names and versions

Excel or cvs

### 3.5 Language

American English

### 3.6 License

CC0 1.0

### 3.7 Limits to Sharing

None

### 3.8 Publication date

May 29, 2025

### 3.9 FAIR data/Codebook

In the dataset, the demographic variables were coded as follows: age (range: 18–28), major (STEM = 1; non-STEM = 0), high school GPA (hsGPA; range: 0–100), and first-year GPA of university studies (fyGPA; range 0–4). Self-report variables were coded as follows: PRMQ (range: 1–5), GAD-7 (severity items’ range: 0–3; impact range: 0–3), and GASE (range: 1–5). A readMe file is included.

## (4) Reuse potential

The current dataset can be used for cross-cultural and educational research, comparing and contrasting different first-year student populations (e.g., STEM and non-STEM majors; Pilotti et al., 2024). Machine learning methods, which often serve as an important tool for data mining devoted to performance predictions, can also benefit from benchmark datasets, such as ours (Chen & Zhai, 2023). Validation studies can utilize the dataset to assess the suitability and generalizability of particular self-report measures (Arafat et al., 2016). Lastly, the current data represent a capsule of how young women respond to societal changes on top of changes in their everyday lives brought about by academic demands. As such, these data may add to research concerned with learners’ responses to change (De la Sablonniere, 2017) if additional data from Saudi Arabian learners at higher levels of their undergraduate educational journey are used for comparison.
