# Supplementary material for: Data From the File Drawer: Bridging the Data Gap About Female First-Year Learners’ Performance and Psychological Well-Being in a Bilingual Context
Source: J Open Psychol Data. 2026 Sep 11;14:3. doi: 10.5334/jopd.141 (PMC13564939; doi:10.5334/jopd.141)
Supplement: Supplementary file 1 [file jopd-14-141-pr1.pdf]

Initial Peer Review Round for "Data from the File Drawer: Bridging the data gap about female freshmen’s performance and psychological well-being"

Dear Maura Pilotti, Maryam Bo Julaia , Arifi Waked , Omar El-Moussa ,

After review, we have reached a decision regarding your submission to Journal of Open Psychology Data, "Data from the File Drawer: Bridging the data gap about female freshmen’s performance and psychological well-being". Our decision is to request revisions of the manuscript prior to acceptance for publication.

The full review information is included at the bottom of this email. Please note that there may also be a copy of the manuscript file with reviewer comments available once you have accessed the submission account. In summary, we all recognise great value in the data collected, but would like a little more detail and specificity in the write-up to maximise its potential for reuse.

Instructions for how to resubmit your article online are pasted below. Please ensure that your revised files adhere to our author guidelines, and that the files are fully proofed prior to upload. Please also include a revised version of your article with 'tracked changes', adding comments where appropriate, to indicate the revisions made, in addition to a brief document outlining how you have responded to the reviewers' requests.

If you have trouble processing the revisions, our Help Center (https://help.u-community.io) or downloadable PDF (https://bit.ly/Author-Guide-OJS-3) may be able to help. If not, please get in touch and we’ll be happy to help.

Please also ensure that all copyright permissions have been attained for any figures/tables you have included.

Please could you have the revisions submitted with two weeks. If you cannot make this deadline, please let us know as early as possible.

Kind regards,

Prof Thomas Rhys Evans

Reviewer 1:
Recommendation: Revisions Required

Comments to the author(s)

The submitted manuscript is a short data paper describing a dataset including student grades and self-report measures on memory concerns, anxiety symptoms and academic confidence levels. It presents an impressive sample of 1141 participants. It is generally well written but lacks important information if the dataset should be reused in the future. I provide a list of comments outlining where clarifications are needed. All in all, this is an interesting submission and will make a good contribution to the journal.

Submission requirements according to the review guidelines:
• The paper content requirements have been mostly met, except for the requested clarifications below
• The deposited data requirements have been mostly met. The following should be addressed:
o The shared datafile has the .xlsx extension, which is a proprietary software data file, please share it in a non-proprietary format, for example, .csv file
o There is no codebook, please share a readme.txt or a similar non-proprietary file format with a description of all data – including the operationalisation of columns/variables – i.e. how is high-school GPA operationalised and how it differs from the university GPA

Comments – Abstract:
- I am a bit unsure about your last sentence – “Data can contribute to the development of prediction models inclusive of human diversity, and, more broadly, to cross-cultural research.” You do not mention such type of research anywhere in your introduction, so it does not feel like this is what the data were collected for in the first place. I would replace this sentence with a statement that is more closely aligned with the original aim of your project.
Comments – Background:
- It seems like there is a lot of information missing after the second paragraph and before the last paragraph. In paragraph 2, you describe some important context for your study and outline the topic – the experiences of female students attending their first year of university education. After this paragraph, the aim of the study should be clearly described. What is the purpose of researching this topic? You should clearly describe the aims of your project to give a clear rationale for how the study was designed. Was the aim to gain a new understanding in a specific area of educational or social psychology? Or to explore whether the students face difficulties and how they can be better supported? This is very important for the reader to better understand the motivation behind the decisions pertaining to the design of your study. It will also be very helpful when reusing the data in the future. Especially if

your data should be used together with data collected elsewhere and for projects with different or similar initial aims.
- Paragraph 3 – GPA acronym must be written out in full when mentioned for the first time
- Paragraph 3 – should give a clearer summary about the study. It should also link directly to the project aims. State that data were collected using self-report assessments and state what was assessed exactly. Currently you mention “offers a snapshot of students’ confidence in their abilities”, “emotional difficulties” and “cognitive challenges” but you can be more concrete and say that participants completed 3 questionnaires, each assessing a different construct – anxiety symptoms, memory concerns and academic confidence. Or something along these lines. It is important that it becomes clear why these specific constructs and questionnaires were chosen for your study.

Comments – Method:
- The study design section is unclear to me. Does ‘freshmen’ mean first year undergraduate/bachelor students at a university? If yes, then what is a “general education curriculum” at a university? I am not familiar with such a concept – what is it that these students were studying? Or how is the “general education curriculum” relevant here? Perhaps this context should be explained in the introduction.

- Sampling, sample, and data collection
o How did you check if participants were of Saudi Arabian descent and completed their previous education in the country?
o How did you ask participants to take part in your study? Or how did you advertise? Through email, in-person during workshops, through teachers?
o What is the 97% participation rate? Does it mean you invited an X number of participants and 97% responded to your advertisement? Then, specify how many you invited, how many responded and how many completed the whole study and were included in the dataset.
o Provide the range and mean age for your sample.
o In your data, I see that the lowest high school grade is 63.4 and the average is 94.59. What does this say about your sample? Are they all top performing high school graduates?
o Include more information here - did you test the participants face to face? Did they complete the questionnaires by themselves or did you read the questions? Did they complete the questionnaires on paper or electronically? If electronically, what software was used to display the questions? Were all questionnaires completed in one go? How long did it take participants to complete the study? Was there any reimbursement for participation?

- Materials/ Survey Instruments
o Currently, paragraph 1 is more suitable for section 3.9. In this section, you should

instead describe your demographic questionnaire. Specify what demographic information was collected from participants and why. How did you decide what questions to ask to meet the aims of your project?

o How are GPA points operationalised exactly? I am curious to know how you found these numbers so precisely with these decimal points. This should be explained for both the high school and university grades. The calculation and interpretation of these grades should be very clear to the reader.

o Provide full names for each measure/questionnaire used. If applicable, also specify the version of the measure.

o Explain how the scores are interpreted - is there a total score calculated for each of the measures? Are there any items with reverse scoring? What about the total score for subscales? Does the total score have any interpretation depending on different thresholds? For example, clinical anxiety levels. The total scores are not included in your data file, which is ok, but the future user of your data should know how they can calculate meaningful variables.

o Specify in what language the tests were completed.

- Quality Control

o “Each item was rated on a 5-point scale from relevant (+2) to irrelevant (-2) to the construct intended to be tested.” You should explain what you mean by this exactly. What constructs? Could you share the exact questions you administered to the participants to test this?

o Would it be possible to share the data for these assessments? If not, why?

o You could add an additional comment about how your validation ensured that these measures were suitable for implementation in the specific context of your study. Because these measures have been validated before, but your context is different compared to the previous validations.

- Data anonymization and ethical issues

o AP guidelines – the acronym needs to be written out in full

o Specify the exact ethics review board including the full name of the institution

- Reuse potential

o In your last sentence, you write: “As such, these data may add to research concerned with people’s responses to change (De la Sablonniere, 2017).”

I am not convinced that your data are well fitted to measure response to change. You have cross-sectional data collected at one time-point. A dataset suitable to investigate a response to change would include dependent/outcome variables with data recorded at several time points in relation to a particular event.

Reviewer 2:

Recommendation: Revisions Required

Comments to the author(s)

"Data from the File Drawer: Bridging the data gap about female freshmen's performance and psychological well-being,"

Submitted by: Pilotti, Maura (Prince Mohammad bin Fahd University)

Review of the Data Paper

This is a well-executed data paper that clearly justifies its purpose of making an unpublished dataset available for future scientific use. I particularly appreciated the paper's premise that "findings of educational science are too often defined by the contribution of student populations of the Global North." By focusing on Arabic women students, the dataset contributes meaningfully to addressing this imbalance and has strong potential value for the research community.

Methods Used to Create the Dataset:

The dataset is well structured and includes a solid range of independent and dependent variables. It demonstrates high reuse potential, particularly for research comparing Arabic women in STEM versus non-STEM programs (e.g. Haddad et al. (2025); Pilotti et al. (2024)). This dataset is also relevant for studies evaluating academic performance of students in undergraduate degree programs (e.g., Tewari, 2014). However, to enable robust cross-cultural comparisons (e.g., Global South vs. Global North), data collection could have incorporated additional cultural variables, such as Hofstede's cultural dimensions e.g., masculinity index (Hofstede & Bond (1984)). Also, to improve generalizability authors could have expanded beyond the current sample to include women in additional STEM fields (e.g., Medicine, Biotechnology, Data Analytics) and non-STEM fields (e.g., History, Fine Arts, Community Sciences). These points may be taken into consideration in future research on this topic.

Accessibility and Transparency of the Data:

The dataset is highly accessible and easy to download, with a smooth repository link. I was able to access, examine, and conduct exploratory analyses without difficulty, and the data appear to be transparently reported. While the data dictionary is provided in the paper, including it directly within the data file would significantly improve usability and facilitate variable cross-referencing.

The data appears complete, with no missing values, which raises the question of whether the shared file represents raw or cleaned data or whether all the questions asked in the survey were mandatory. Clarification on this point would enhance transparency. Additionally, including the link to the actual online survey instrument and specifying the survey administration dates would be beneficial.

That said, more detail is needed regarding the mode of data collection. It is unclear whether the data were collected via online surveys or in controlled, in-person

settings, whether respondents completed the survey simultaneously, or whether data collection occurred in multiple phases. Including variables related to family structure or levels of patriarchy could also have enriched the dataset.

The sample size (N = 1141) is adequate. However, given that first-year GPA data are available, the participants appear to be sophomore students rather than freshmen. The reporting of the test–retest reliability and face validity is thorough and well done.

Study Design

The authors describe the study as cross-sectional, implying data collection at a single point in time. However, clarification is needed regarding when the first-year GPA data were obtained relative to the survey administration. It is also unclear whether GPA data were self-reported or retrieved from institutional academic records.

Given that data collection spanned more than 1.5 years, more detailed information about the specific phases and timing of data collection would be valuable. Similarly, specifying the exact institution(s) involved—rather than broadly stating “Saudi Arabia, Eastern Province”—would enhance methodological transparency.

Purposive sampling was appropriate given the study’s targeted population and shared demographic background (age, gender, socio-cultural characteristics). Nevertheless, this sampling approach introduces the risk of bias, and the findings should not be overgeneralized to the broader population of Arabic women students.

Because first-year GPA largely reflects general education courses common across majors, its suitability for comparing academic performance between STEM and non-STEM students is somewhat limited. Additionally, given that participants are Arabic–English bilingual women, the paper’s title and scope should more explicitly reflect this specificity.

Future research can incorporate richer contextual data, including the socio-cultural and family backgrounds of English-speaking and Arabic-only students. Additionally, examining contextual factors such as students’ living arrangements (e.g., residing with family versus in college housing) could provide deeper insights and strengthen the dataset for future research.

Quality Control and Validation

I appreciated the authors’ use of the test–retest reliability and pilot testing to establish face validity for the self-reported measures. However, it would be useful to clarify whether participants from the reliability (n = 35) and validity (n = 25) sub-samples were re-included in the main dataset.

Overall Assessment

Overall, this is a strong and valuable data paper with good data collection methodology and considerable potential for reuse. With additional clarification on

data collection procedures, sampling limitations, the dataset’s transparency, interpretability, and cross-cultural applicability could be enhanced.

References

Haddad, T., BouJaoude, S., Mostapha, L., & Abou Habib, L. (2025). Women and STEM Education in the Arab World. In Cross-Cultural Comparisons of Science Education (pp. 95-124). IGI Global Scientific Publishing.

Hofstede, G., & Bond, M. H. (1984). Hofstede's culture dimensions: An independent validation using Rokeach's value survey. Journal of cross-cultural psychology, 15(4), 417-433.

Pilotti, M. A., El Alaoui, K., Abdelsalam, H. M., & El-Moussa, O. J. (2024). Understanding STEM and non-STEM female freshmen in the Middle East: a post-pandemic case study. Cogent Education, 11(1), 2304365.

Tewari, G. (2014). Academic Performance of the Students (APS) in Undergraduate Degree Program: Perception of Stakeholders at University of Agricultural Sciences, Bangalore (Doctoral dissertation, University of Agricultural Sciences, GKVK).

Reviewer 3:
Recommendation: Revisions Required

Comments to the author(s)

This data paper presents a well-documented dataset addressing an underrepresented population in educational and psychological research. The manuscript largely satisfies the requirements for a JOPD data paper. The methods and dataset description are clear, ethical considerations are adequately addressed, and the data are openly archived under an appropriate license.

However, we request clarification on the following minor points.

1. Regarding item-level handling

While the description of the psychological scales is clear, the manuscript could further clarify whether any items were reverse-coded and whether composite scores were computed or left at the item level in the deposited dataset.

2. Regarding the handling of missing data

It may be useful to explicitly state how missing data (if any) were handled prior to archiving.

3. Regarding the analysis conducted for retest reliability.

The manuscript reports test–retest reliability values ranging from 97% to 98%; however, it is unclear which statistical index was used to compute these values (e.g., Pearson correlation, intraclass correlation coefficient, or percent agreement). Please clarify the exact method used to assess test–retest reliability and report the corresponding statistic using standard notation.

4. Regarding the indicators used for inter-rater agreement rates

While inter-rater agreement was reported, the use of simple agreement percentages may overestimate true agreement.

Given the ordinal rating scale and the number of raters involved, the use of an intraclass correlation coefficient (ICC) or a weighted kappa statistic would provide a more appropriate and informative estimate of inter-rater agreement.

5. Regarding reusability for LLM

The statement that “The coding capabilities of large language models (LLMs) devoted to performance predictions can also benefit from benchmark datasets, such as ours” is conceptually interesting but currently underspecified. Given that the dataset consists entirely of structured, quantitative variables rather than natural language data, it is not immediately clear how LLMs would directly benefit from this dataset. Clarifying this point would strengthen the reuse section and help readers better understand the intended scope of this claim.

6. Regarding the creation of READ ME files

To further enhance third-party usability, the authors might consider adding a brief README file in the repository that mirrors the variable descriptions provided in Section 3.9 of the manuscript.
